# Therapeutic Role of Inducible Nitric Oxide Synthase Expressing Myeloid-Derived Suppressor Cells in Acetaminophen-Induced Murine Liver Failure

**DOI:** 10.3389/fimmu.2020.574839

**Published:** 2020-10-29

**Authors:** Chen-Yu Hsu, Yung-Chang Lin, Li-Yuan Chang, Sheng-Kai Huang, Chien-Hao Huang, Chan-Keng Yang, Ching-Tai Huang, Chun-Yen Lin

**Affiliations:** ^1^ Graduate Institute of Biomedical Sciences, Chang Gung University, Taoyuan, Taiwan; ^2^ Department of Hepatogastroenterology, Chang Gung Memorial Hospital, Taoyuan, Taiwan; ^3^ School of Medicine, Chang Gung University, Taoyuan, Taiwan; ^4^ Division of Medical Oncology/Hematology, Chang Gung Memorial Hospital, Taoyuan, Taiwan; ^5^ Division of Infectious Diseases, Chang Gung Memorial Hospital, Taoyuan, Taiwan

**Keywords:** APAP, MDSC, iNOS, cell therapy, acute liver failure

## Abstract

**Background:**

Acetaminophen (APAP) overdose is one of the major etiologies of liver failure. Hepatocyte necrosis induced by toxic metabolites of APAP can activate proinflammatory responses, including elastase-expressing neutrophils, to exacerbate liver injury. Myeloid-derived suppressor cells (MDSCs) increased in inflammation can inhibit proinflammatory responses. Our aim is to investigate the role of MDSC in APAP-induced liver failure and the possible therapeutic application.

**Methods:**

BLAB/c mice were injected with a sublethal/lethal dose of APAP as the murine model of liver failure. MDSCs were defined as CD11b^+^Gr-1^+^ cells with the ability of T-cell suppression.

**Results:**

A sublethal challenge of APAP could increase the intrahepatic MDSC and protect mice against subsequent lethal challenge of APAP, lipopolysaccharide (LPS)/D-galatosamine or concanavalin A. This protection was lost if MDSCs were depleted and inducible nitric oxide synthase (iNOS) was the key molecule in this MDSC-mediated protection. Taking advantage of these observations, different bone marrow-derived MDSCs (BM-MDSCs) were generated. Among different cytokine-treated BM-MDSCs, tumor necrosis factor alpha/LPS-primed MDSCs (TNF-α/LPS MDSCs) had the strongest liver-protection ability after adoptive transfer. Further mechanistic explorations showed, iNOS-expressing TNF-α/LPS MDSCs induced the apoptosis of activated neutrophil and decreased the intrahepatic infiltration of elastase-expressing neutrophil. Moreover, we generated MDSCs from human peripheral blood mononuclear cells (PBMCs) with similar phenotype.

**Conclusion:**

We demonstrated the protective role of MDSCs and therapeutic effect of TNF-α/LPS MDSCs in APAP-induced liver failure. MDSC might protect against the APAP-induced liver failure by reducing the intrahepatic infiltration of activated neutrophil to limit inflammation. Therefore, a therapeutic role of MDSCs for APAP-induced liver failure was proposed.

## Introduction

Acetaminophen (N-acetyl-p-aminophenol or APAP) is a widely used antipyretic and analgesic drug. However, APAP overdose is also the most common etiology of drug-induced liver injury in the U.S. and Europe with no apparent decline in the past 20 years (1, 2). The clinically available antidote for APAP-induced liver failure is N-acetylcysteine, but N-acetylcysteine is only effective within the initial 8 h after APAP overdose, and gives marginal benefit in clinical practice (1–3). Therefore, there is still an urgent need for new treatments for APAP-induced liver failure.

The pathogenesis of APAP-induced liver failure includes APAP-induced hepatocyte necrosis and the subsequent inflammatory responses. *N*-acetyl-*p*-benzoquinone imine (NAPQI) is the toxic metabolite of APAP that leads to hepatocyte necrosis (4). Danger-associated molecular pattern (DAMP) released by extensive necrotic hepatocytes activates Kupffer cells to secrete inflammatory cytokines and chemokines to recruit monocytes and neutrophils to the necrotic site, and amplifies the inflammatory responses (5–9). Neutrophils are one of the most abundant immune cells in the initial necroinflammatory phase in APAP-induced liver injury (9, 10). High mobility group box 1 (HMGB-1) and mitochondrial products, such as formyl peptides and mitochondrial DNA, can activate and promote the migration of neutrophils to the liver (6, 11, 12). Moreover, activated neutrophil could aggravate liver damage was proved in mice by adoptive transfer (12, 13). Previous study also indicated that neutrophils could amplify liver injury by neutrophil elastase (6). Similarly, the importance of neutrophil elastase had been shown that neutrophil elastase inhibitor, sivelestat, can also reduce the severity of APAP-induced liver failure (14). Therefore, regulating inflammatory responses focusing on neutrophils or monocytes/macrophages could be a way to reduce hepatotoxicity and mortality induced by APAP overdose.

Myeloid-derived suppressor cells (MDSCs) are heterogeneous immature myeloid cells that markedly increase in various inflammatory diseases, such as cancer and autoimmune diseases (15–18). In mice, CD11b^+^Gr-1^+^ cells are identified as total MDSCs including both polymorphonuclear MDSC (PMN-MDSC) and monocytic MDSC (M-MDSC) (19). The main feature of MDSCs is immune suppression. MDSCs can suppress the activation and proliferation of both CD4^+^ and CD8^+^ T cells through the depletion of L-arginine by arginase 1 and promoting the expansion of regulatory T cells by IL-10 and TGF-β production (20). Besides, MDSCs can also regulate innate immune responses, like inhibits the activation of natural killer cell by the engagement of NKp30 receptor (21). Taking the advantages of immune regulatory abilities of MDSCs, the therapeutic roles of MDSCs in inflammatory diseases had been explored. For example, the adoptive transfer of MDSCs generated from bone marrow cells could benefit the islet transplantation and graft-versus-host disease in several animal models (22–25).

Based on the above evidence, we hypothesized that MDSCs might play a therapeutic role in APAP-induced liver injury (AILI). Therefore, we set up the AILI animal model and investigated the possible role of MDSCs and explored the therapeutic aspect of *in vitro*-generated MDSCs in AILI.

## Materials and Methods

### Ethics Approval Statement

All animal studies were carried out in accordance with guidelines approved by the institutional animal ethics committee of the laboratory animal center of Chang Gung Medical Foundation (protocol # 2010090802; 2016092101). Studies with human peripheral blood mononuclear cells were carried out in accordance with guidelines approved by Chang Gung Medical Foundation Institutional Review Board (protocol # 201801460A3), and all subjects gave written informed consent according to the guidelines established by Chang Gung Medical Foundation Institutional Review Board.

### Experimental Animals

Male BALB/c mice aged 8–10 weeks were purchased from national laboratory animal center (Taipei, Taiwan) for experiments. iNOS knockout mice (Stock No: 007072) on BALB/c background were obtained from The Jackson Laboratory. All animal breeding and experiments were in accordance with guidelines approved by the institutional animal ethics committee of the laboratory animal center of Chang Gung Medical Foundation (Taoyuan, Taiwan).

### APAP, Con A, LPS/D-GalN, and N-Acetylcysteine Treatment

APAP (Cat# A7085), lipopolysaccharide (LPS, Cat# L4391), D-galatosamine (D-GalN, Cat# G0500), concanavalin A (Con A, Cat# C0412), and N-acetylcysteine (Cat# A9165) were purchased from Sigma-Aldrich and dissolved in phosphate-buffered saline respectively. Because female mice had a lower susceptibility to APAP hepatotoxicity (26), all mice used in this study are male mice to reduce the variance. All mice were fasted overnight before the intraperitoneal injection of APAP (200 mg/kg or 400 mg/kg) or LPS (10 μg/kg)/D-GalN (400 mg/kg), and Con A (15 mg/kg) was intravenously injected into mice without fasting. For the treatment of antidote, N-acetylcysteine (300 mg/kg) was intraperitoneally injected into mice. Each experimental group contains 3–6 mice in each individual experiment.

### Assessment of Hepatotoxicity

The serum levels of alanine aminotransferase (ALT) and aspartate aminotransferase (AST) were determined by ALT GPT Colorimetric Method (BIOLABO Cat# 92027) and FUJI DRI-CHEM SLIDE GOT/AST-P III (FUJIFILM, CODE#3150) using DRI-CHEM NX500i system (FUJIFILM) respectively.

### Histology

For histological analysis, mice were sacrificed at 12 h after APAP injection, and liver tissues were fixed with formalin and embedded with paraffin. Liver sections were cut at 5 μm and stained with hemotoxylin and eosin. The quantification of liver necrosis was calculated using ImageJ (National Institute of Mental Health, Bethesda, Maryland, USA).

### Antibodies and Flow Cytometry

For flow cytometry analysis, hepatic leukocytes, splenocytes, bone marrow cells, and blood leukocytes were isolated. The isolation of liver leukocytes was described previously (27). Briefly, the liver tissue was minced in Hanks’ balanced salt solution (GIBCO Cat# 14025126) and passed through sterile gauze and a cell strainer to obtain a single-cell suspension. The cell suspension was centrifuged at 500 g for 5 min, and the cell pellet was resuspended in 15 ml of 33% Percoll (GE Healthcare Cat# 17089101) containing 100 U/ml of heparin (Sigma-Aldrich Cat# H3149) and centrifuged at 500 g for 15 min. The disrupted hepatocytes and debris on the top layer of the supernatant was discarded, and the cell pellet containing leukocytes was resuspended in red blood cell lysis solution and washed twice in RPMI 1640 (GIBCO Cat# 31800089) containing 5% fetal bovine serum. The spleen was minced in RPMI-1640 and passed through a cell strainer to obtain a single-cell suspension. The splenocytes were acquired after the lysis of red blood cells. The bone marrow cells were flushed with RPMI-1640 from the femurs and tibiae of mice. The erythrocytes were lysed and the bone marrow cells were washed twice in RPMI-1640. The blood leukocytes were obtained after the lysis of red blood cells. ACK (Ammonium-Chloride-Potassium) lysing buffer was used for the lysis of red blood cells.

The single-cell suspensions were incubated with antibodies as per manufacturer’s instruction, acquired by FACSCanto II (BD Bioscience), and analyzed using FlowJo software v7.6. The following antibodies were used for flow cytometry staining: FITC-conjugated anti-Gr-1 (BD Biosciences Cat# 553126, RRID:AB_394642), anti-Ly6G (BD Biosciences Cat# 551460, RRID:AB_394207) and anti-iNOS (BD Biosciences Cat# 610330, RRID:AB_397720), PreCP/Cy5.5-conjugated anti-Gr-1 (BD Biosciences Cat# 552093, RRID:AB_394334) and anti-CD45 (BioLegend Cat# 103132, RRID:AB_893340), and APC-conjugated anti-CD11b (Thermo Fisher Scientific Cat# 17-0112-83, RRID:AB_469344) antibodies were used for mouse cell staining. The absolute cell number was calculated by multiplying the percentage of each population from 2-parameter flow cytometry data by the total number of isolated leukocytes per liver. Human MDSCs were defined by the staining of PerCP/Cy5.5-conjugated anti-human CD33 (BioLegend Cat# 366616, RRID:AB_2566418), and PE-conjugated anti-human CD11b (BD Biosciences Cat# 557321, RRID:AB_396636) antibodies.

### Bone Marrow-Derived MDSC Culture

2×10^6^ bone marrow cells were seeded in 12-well plates with 2 ml of RPMI-1640 containing 10% FBS, 10 ng/ml granulocyte-macrophage colony-stimulating factor (GM-CSF, Cat# 315-03) or combined with 50 ng/ml interleukin-6 (IL-6, Cat# 216-16) or tumor necrosis factor alpha (TNF-α, Cat# 315-01A). For cell activation, LPS (100 ng/ml) was added on day 3. On day 4, MDSCs were harvested as non-adherent cells and washed twice with HBSS to reduce the contamination of LPS and cytokines. All recombinant proteins were purchased from PeproTech.

### T Cell Suppression Assay

CD11b^+^Gr-1^+^ cells were sorted by FACSaria (BD Bioscience) and cultured with Violet Proliferation Dye 450 (BD Horizon Cat# 562158)-labeling CD8^+^ T cells (1×10^5^) with stimulation by anti-CD3 (BD Biosciences Cat# 553057, RRID:AB_394590) and CD28 antibodies (BD Biosciences Cat# 553294, RRID:AB_394763) for 3 days. Each culture was analyzed by FACSCanto II, and the division index of CD8^+^ T cells was calculated by FlowJo v10.5.3. Results were presented as percent suppression:

$$\text{Percent suppression}=100-\frac{Division\;Index\;of\;\text{T cells}\;with\;MDSC\;present}{Division\;Index\;of\;\text{T cells}\;without\;MDSC\;present}\times 100$$

### Total RNA Extraction and Real-Time PCR

Total RNA was extracted from BM-MDSC using TRIzol (Invitrogen Cat# 15596026), and cDNA was synthesized using MMLV reverse transcriptase (Epicentre Cat# RT80125K). Real-time PCR was performed with iQ SYBR Green Supermix (Bio-Rad) using CFX96 Real-Time PCR Detection System (Bio-Rad). The relative gene expression of each target genes in BM-MDSCs was calculated following the 2 ^−ΔΔCT^ Method (28). The primer sequences for murine MDSC were described in **Supplementary Table**. For the real-time PCR of human MDSC, total RNA of CD33^+^ MDSCs was purified, and the primer sequences were described previously (29).

### MDSC Depletion

To deplete MDSC *in vivo*, 100 μg of anti-mouse Ly6G/Ly6C (Gr-1) antibody (Bio X Cell Cat# BE0075, RRID:AB_10312146) were intraperitoneally injected into mice. The control group is the mice received rat IgG2b antibody (Bio X Cell Cat# BE0090, RRID:AB_1107780).

### Arginase *In vivo* Depletion

Mice were intraperitoneally injected with nor-NOHA (Cayman Cat# 10006861) at a dose of 40 mg/kg 15 min before the lethal challenge of APAP (800 mg/kg) to deplete arginase *in vivo.*

### iNOS Activity

The culture media of BM-MDSC were replaced with Dulbecco’s Modified Eagle Media (Gibco Cat# 12100046) on day 3 of the culture to reduce the high nitrate levels in the RPMI 1640 media. On day 4, the culture media were collected to measure total nitrate/nitrite by the Nitrate/Nitrite Colorimetric Assay Kit (Cayman Cat# 780001).

### *In vitro* Generation of Human MDSC and Suppression Assay

PBMCs isolated from healthy donors were incubated with 10 ng/ml GM-CSF (Peprotech Cat# 300-03) alone or combined with 10 ng/ml TNF-α (Peprotech Cat# 300-01A) in RPMI 1640 media containing 10% FBS. For the culture of TNF-α/LPS MDSC, LPS (100 ng/ml) was added on day 3. Both non-adherent and adherent cells were collected on day 4. The percentages of MDSCs were defined by the staining of anti-human CD33 (BioLegend Cat# 366616, RRID:AB_2566418) and CD11b (BD Biosciences Cat# 557321, RRID:AB_396636) antibodies.

For the suppression assay, CD33^+^ cells were sorted by FACSaria, and cultured with the autologous CD3^+^ T cells purified from the PBMC using Human T Cell Enrichment Kit (STEMCELL Cat# 19051). The proliferation of Violet Proliferation Dye 450-labeling T cells (1 x 10^5^ cells) stimulated by anti-CD3 (BioLegend Cat# 317302, RRID:AB_571927) and anti-CD28 antibodies (BioLegend Cat# 302902, RRID:AB_314304) for 3 days was analyzed by FACSCanto II.

For studies of human MDSCs, the permission from Chang Gung Medical Foundation Institutional Review Board was obtained, and a total of 5 donors was used.

### Neutrophil Elastase Measurement

Hepatic CD11b^+^Ly6G^+^ neutrophils were sorted by FACSaria II (BD Bioscience), and the cell extract was collected. The concentration of elastase was measured by Mouse Neutrophil Elastase SimpleStep ELISA Kit (Abcam Cat# ab252356).

### Total Reactive Oxygen Species (ROS)/Reactive Nitrogen Species (RNS) Measurement

Six hours after sublethal APAP injection, mice were sacrificed and total ROS/RNS levels in the liver tissue homogenate were measured by OxiSelect *In Vitro* ROS/RNS Assay Kit (CELL BIOLABS Cat# STA-347).

### Assessment of Neutrophil Apoptosis

Blood neutrophils were separated by Ficoll-Paque (GE Healthcare Cat# 17144003), followed by hypotonic lysis of red blood cells and stained with FITC-conjugated anti-Ly6G antibody for FITC positive selection (STEMCELL Cat. 17668). The purity of purified Ly6G^+^ neutrophil was more than 95%. For neutrophil activation, 50 nM of phorbol 12-myristate 13-acetate (Sigma-Aldrich Cat# P8139), was added to the culture. Neutrophils were stained with PE Annexin V and 7-AAD (BD Pharmingen Cat# 559763) to evaluate the apoptosis induced by MDSC or DPTA NONOate (Dipropylenetriamine NONOate, Abcam Cat# ab145198), the nitric oxide donor.

### Statistical Analysis

All data were expressed as mean ± SD. For statistical analysis of differences between two groups, Mann-Whitney test was used. Kruskal-Wallis test with Dunn’s multiple comparison test were performed to test more than 2 non-parametric groups. Log-rank test was used for survival analysis between groups. The calculations were made using PRISM version 5.00 (GraphPad Software).

## Results

### Hepatic MDSC Increased After APAP-Induced Liver Injury

To investigate the possible underlying immune mechanisms in APAP-induced liver injury (AILI), we focused on the role of MDSCs, due to their significant increase and regulation of immune responses in several inflammatory conditions (15, 18). Murine MDSCs were defined by the co-expression of CD11b and Gr-1 with the *in vitro* immunosuppressive ability. We induced acute liver injury in mice by challenging a sublethal dose (200 mg/kg) of APAP. The serum ALT levels peaked at 24 h, and returned to the normal range on day 7 after APAP injection (**Supplementary Figure 1A**).

**Figure 1:**
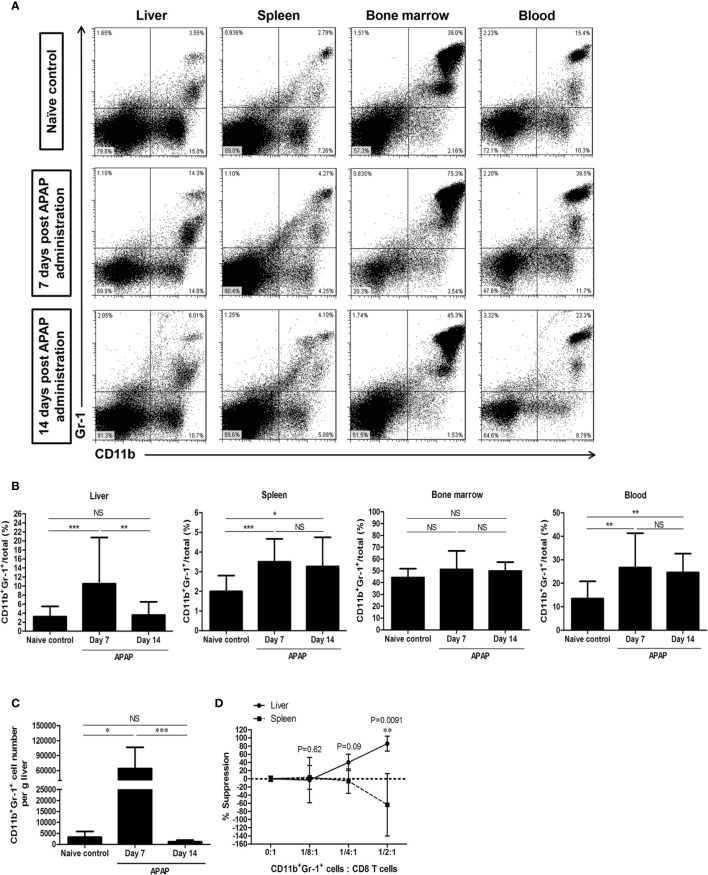
CD11b^+^Gr-1^+^ myeloid-derived suppressor cells (MDSCs) increase in the liver after sublethal acetaminophen (APAP) challenge. **(A)** Representative staining of CD11b^+^Gr-1^+^ cells. **(B)** The frequencies of CD11b^+^Gr-1^+^ cells in different compartments enumerated at 7 days or 14 days after APAP treatment. Naïve control group was healthy mice without any treatment (n=10-18 per group). *P < 0.05, **P < 0.01, ***P < 0.001. Kruskal-Wallis test. **(C)** Absolute cell numbers of CD11b^+^Gr-1^+^ cells in the liver. *P < 0.05, ***P < 0.001, NS, no significance, Kruskal-Wallis test. **(D)** The suppressive abilities of CD11b^+^Gr-1^+^ cells sorted either from the liver or the spleen at 7 days after APAP pretreatment, Mann-Whitney test. Data were expressed as mean ± SD.

Although the liver injury was reduced and the serum ALT levels were in the normal range on day 7 after sublethal APAP challenge, the frequencies of CD11b^+^Gr-1^+^ cells increased in the liver, and continually increased in the spleen and blood up to 14 days, but did not increase during the whole course in the bone marrow compartment (**Figures 1A, B**). The absolute cell numbers of CD11b^+^Gr-1^+^ cells in the liver also significantly increased on day 7 after sublethal APAP challenge (**Figure 1C**). We then purified CD11b^+^Gr-1^+^ cells from the liver and spleen on day 7 after APAP challenge and investigated their suppressive abilities. The results showed only CD11b^+^Gr-1^+^ cells from the liver, but not from the spleen, had suppressive ability (**Figure 1D**). These evidences suggested that the AILI induced the accumulation of CD11b^+^Gr-1^+^ MDSCs in the liver.

### A Sublethal Dose of APAP Could Protect Mice From Subsequent Lethal Challenge of APAP, Con A, or LPS

**Figure 2:**
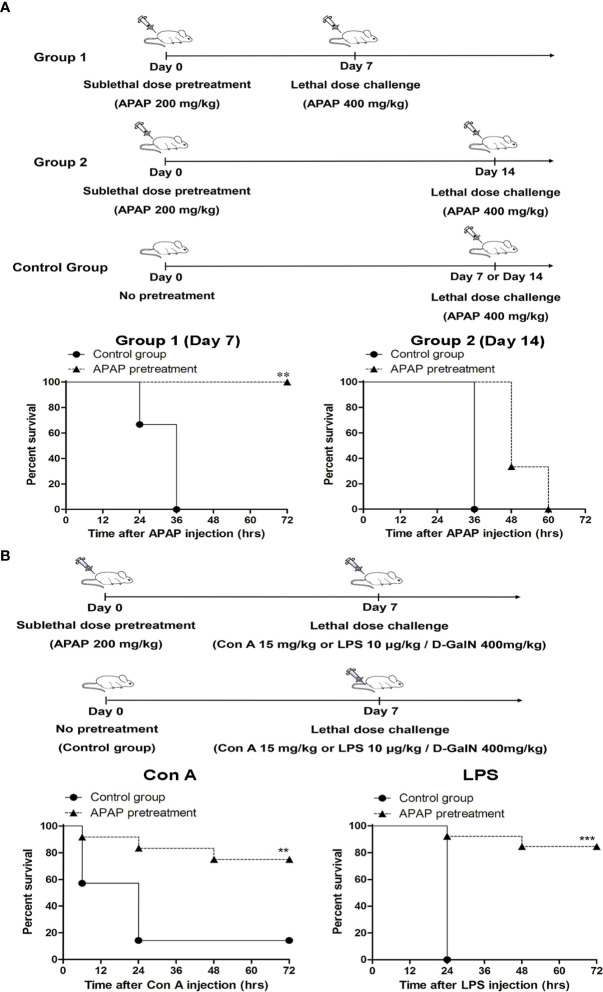
Pretreatment with a sublethal dose of APAP protects mice from subsequent lethal challenge of acetaminophen (APAP), Con A, or lipopolysaccharide (LPS). **(A)** Mice (n=6 per group) were treated with a sublethal dose of APAP (200 mg/kg) on day 0. 7 days (Group 1) or 14 days (Group 2) after, mice were challenged with a lethal dose of APAP (400 mg/kg) respectively. The survival curves were plotted. **(B)** Mice were treated with a sublethal dose of APAP (200 mg/kg) on day 0. 7 days after, mice were challenged either with Con A or LPS/D-GalN (n=12 per group). The survival curves were plotted. The control groups were mice without APAP pretreatment, but received the lethal dose challenge. **P < 0.01, ***P < 0.001, Log-rank test.

To investigate the protective role of MDSC in acute liver failure, we challenged mice with a lethal dose of APAP after sublethal challenge of APAP. Interestingly, these sublethally challenged mice could survive the subsequent lethal dose of APAP (400 mg/kg) challenged on day 7 after sublethal challenge. However, this protection was lost when the lethal dose was challenged on the day 14 after the sublethal challenge (**Figure 2A**). One of the possibilities of this protection was caused by an increase of hepatic glutathione in the liver after sublethal APAP challenge that in turn rapid metabolized the excess amount of highly reactive toxic intermediate, NAPQI, of APAP by conjugation (30). To clarify this issue, we examined the levels of reduced glutathione in the liver of sublethal APAP-treated mice. Compared to naive mice, the levels of reduced glutathione in the liver of APAP-treated mice on day 7 and day 14 post pretreatment did not differ significantly (**Supplementary Figure 1B**). This liver injury-induced protection was not due to the increase of hepatic glutathione.

**Figure 3:**
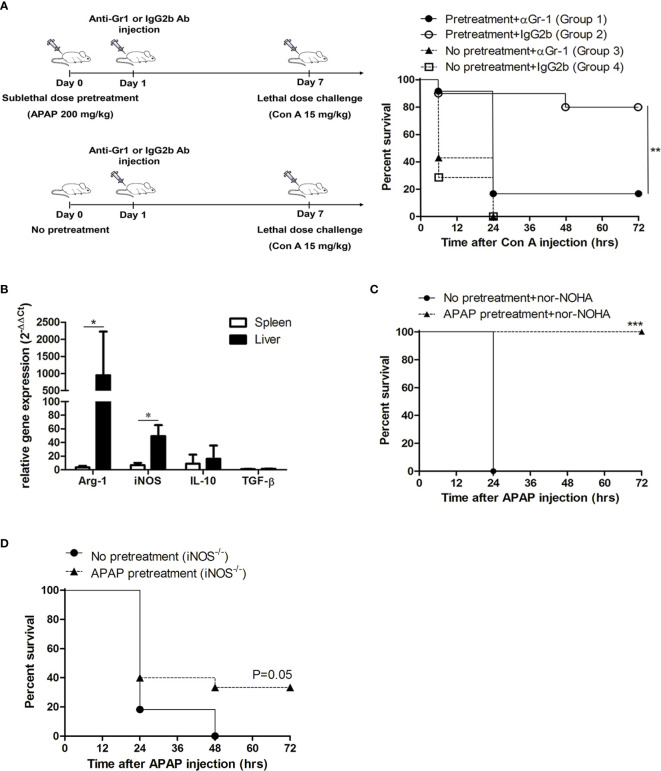
The protective effect induced by acetaminophen (APAP) pretreatment is mediated through myeloid-derived suppressor cell (MDSC) and inducible nitric oxide synthase (iNOS). **(A)** Mice received sublethal APAP pretreatment on day 0 and were injected intraperitoneally with anti-Gr1 antibody (Group 1) or Rat IgG2b isotype antibody (Group 2) on day 1. On day 7, mice were challenged with a lethal dose of Con A. Group 3 and 4 were mice without sublethal APAP pretreatment, but received anti-Gr1 antibody (Group 3) or Rat IgG2b isotype antibody (Group 4) on day 1. On day 7, mice were challenged with a lethal dose of Con A. The survival curves were plotted (n=12 per group). **P < 0.01, Log-rank test. **(B)** mRNA expression of CD11b^+^Gr-1^+^ cells isolated from the liver and the spleen at 7 days after APAP pretreatment. *P < 0.05, Mann-Whitney test. Data were expressed as mean ± SD. **(C)** BALB/c mice with or without APAP pretreatment were injected with nor-NOHA before the lethal dose challenge on day 7 and the survival curves were plotted. (No APAP pretreatment group, n=6; APAP pretreatment group, n=12). ***P < 0.001, Log-rank test. **(D)** iNOS knockout mice with or without APAP pretreatment on day 0 were challenged with a lethal dose of APAP on 7 and the survival curves were plotted. (No APAP pretreatment group, n=11; APAP pretreatment group, n=15). P=0.05, Log-rank test.

Because the pretreatment of APAP might also affect drug metabolism genes that in turn induce signifcant responses to mitigate liver injury (31), we challenged these sublethal APAP-pretreated mice with a lethal dose of Con A or LPS/D-GalN, which can induce immune-mediated liver injury in mice (32, 33), on day 7 after APAP pretreatment. Similar to the results of the lethal dose challenge of APAP, these APAP-pretreated mice could also survive the lethal challenges of Con A and LPS/D-GalN (**Figure 2B**). To further clarify the role of MDSCs in the sublethal APAP-induced protection, MDSCs were depleted by the injection of anti-Gr1 antibody after sublethal APAP challenge. Firstly, the depletion effect of anti-Gr1 antibodies was evaluated. The injection of anti-Gr1 antibodies one day after sublethal APAP challenge could significantly reduce the intrahepatic CD11b^+^Gr-1^+^ cells (**Supplementary Figure 2A**). On day 6, the percentages of CD11b^+^Gr-1^+^ cells were returned to the normal range compared with mice injected with isotype antibody (**Supplementary Figure 2B**). Moreover, the depletion of CD11b^+^Gr-1^+^ cells on day 1 after APAP pretreatment did not affect the liver damage and resolution reflected as serum ALT levels on day 1 and day 7 by sublethal APAP (**Supplementary Figure 2C**). Because the depletion of Gr-1^+^ cells by anti-Gr1 antibody could persist less than 6 days, we challenged the mice with a lethal dose of Con A on day 7. As shown in **Figure 3A**, the protection caused by sublethal APAP pretreatment was lost after MDSC depletion by day 1 anti-Gr-1 antibodies treatment. These results revealed a sublethal challenge of APAP could protect mice from subsequent lethal liver injury, challenged by either APAP, Con A, or LPS/D-GalN, and MDSC is the key player in this sublethal APAP-induced protection.

### iNOS But Not Arginase Played the Key Role in the Protection Effect of Sublethal APAP Pretreatment

To clarify the immune suppressive mechanisms of MDSC in this sublethal APAP-induced protection, we examined the mRNA expression of hepatic MDSC by real-time PCR. The results showed that the mRNA expressions of arginase 1 and iNOS, which were the metabolic enzymes highly expressed in MDSCs, were markedly increased in the CD11b^+^Gr-1^+^ cells isolated from the liver, compared to the spleen (**Figure 3B**). Since the hepatic MDSCs had an elevated expression of arginase 1 and iNOS, we then investigated the role of the two enzymes in the protection effect of sublethal APAP pretreatment. We used arginase inhibitor, nor-NOHA, to reduce liver arginase activity in mice before lethal challenge of APAP after APAP pretreatment (**Supplementary Figure 3**). The results showed the treatment of nor-NOHA could not reduce the protection effect induced by sublethal APAP pretreatment (**Figure 3C**). In contrast, the protection effect of sublethal APAP pretreatment was lost in iNOS knockout mice (**Figure 3D**). These results clearly indicated the liver injury-induced and MDSC-mediated protection was through iNOS but not arginase.

Based on the above evidence, we then explored the possible therapeutic potential of MDSC in the APAP-induced acute liver failure. For this purpose, we focused on the therapeutic role of bone marrow-derived MDSCs (BM-MDSCs) generated by *in vitro* culture.

### Adoptive Transfer With TNF-α/LPS-Primed BM-MDSCs Protected Mice From the Mortality Induced by APAP

**Figure 4:**
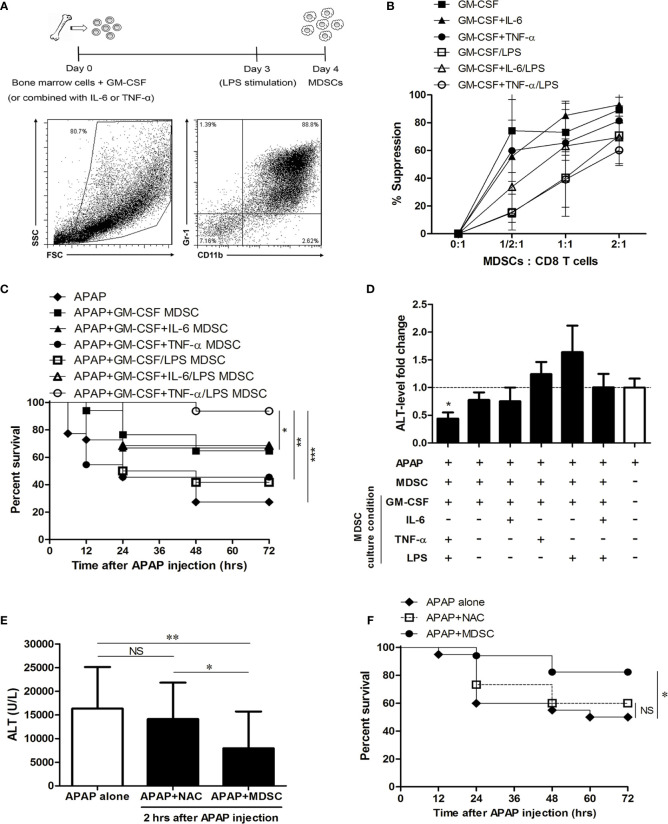
The therapeutic effect of *in vitro*-generated myeloid-derived suppressor cells (MDSCs) in APAP-induced liver injury. **(A)** The experiment scheme of *in vitro*-generated MDSCs. **(B)** The suppressive abilities of MDSCs. These *in vitro*-generated MDSCs (8 x 10^6^ cells) were adoptively transferred into mice just after APAP challenge to evaluate the survival **(C)** and the fold change of ALT level at 12 h after APAP injection **(D)**, n=12-16 per group. Log-rank test and Kruskal-Wallis test are used to analyze survival curve and the fold change of ALT level respectively. *P < 0.05, **P < 0.01, ***P < 0.001. **(E)** The ALT level of the post-treatment of TNF-α/LPS MDSC or NAC at 12 h after APAP injection. At 2 h after APAP injection, TNF-α/LPS MDSCs were adoptively transferred into mice, and NAC was intraperitoneally injected into mice respectively, n=15-20 per group. *P < 0.05, **P < 0.01, NS, no significance, Kruskal-Wallis test. **(F)** The survival of the post-treatment of TNF-α/LPS MDSC and NAC (n=15-20 per group). *P < 0.05, **P < 0.01, ***P < 0.001, NS, no significance, Log-rank test. Data were expressed as mean ± SD.

**Figure 5:**
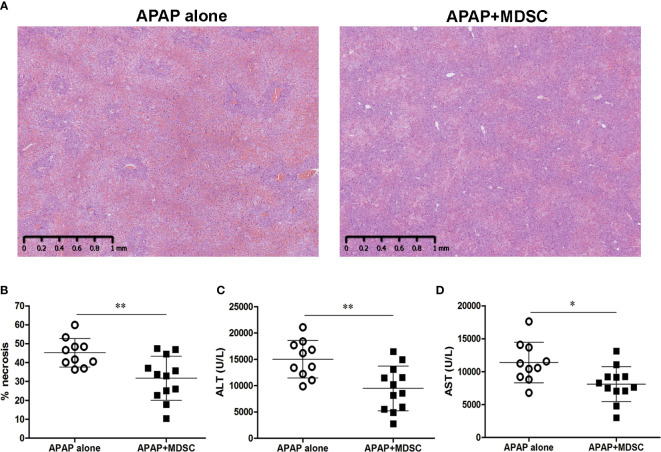
Adpotive transfer of TNF-α/lipopolysaccharide (LPS) myeloid-derived suppressor cells (MDSCs) reduces acetaminophen (APAP)-induced liver injury **(A)** Liver histological stain of mice injected with APAP alone or combined with adoptive transfer of TNF-α/LPS MDSCs. **(B)** The quatification of liver necrosis. **(C)** Serum ALT levels and **(D)** Serum AST levels at 12 h after APAP challenge (n=10-12). *P < 0.05, **P < 0.01, Mann-Whitney test. Data were expressed as mean ± SD.

Because MDSC could be generated *in vitro* from bone marrow cells in the presence of GM-CSF and their immunosuppressive function could be enhanced by pro- inflammatory cytokines/molecules (22, 23, 34), we cultured bone marrow cells with GM-CSF alone or combined with inflammatory cytokines, such as IL-6 and TNF-α, and LPS in order to enhance the suppression abilities of BM-MDSCs. The percentages of CD11b^+^Gr-1^+^ cells were around 85%–90% in the four-day cultures (**Figure 4A**). The suppressive abilities of BM-MDSCs were examined by suppressing CD8 T cell proliferation. The results showed there is no statistically significant difference in the suppressive abilities among different BM-MDSCs (**Figure 4B**). We then adoptively transferred these different BM-MDSCs into mice challenged with sublethal dose of APAP separately to investigate their therapeutic effects. As shown in **Figure 4C**, the *in vitro*-generated MDSCs cultured with TNF-α/LPS (TNF-α/LPS MDSCs) had the most potent ability to rescue mice from mortality compared with other BM-MDSCs. Besides, only TNF-α/LPS MDSCs could significantly reduce the serum ALT levels (**Figure 4D**). To evaluate the protective effect of TNF-α/LPS MDSCs in AILI, the necrotic area of H&E stained liver sections was measured. The hitological analysis indicated the reduced liver injury in the mice with adoptively transfer of TNF-α/LPS MDSCs (**Figures 5A, B**). Similar to the results of hitological analysis, mice with adoptive transfer of TNF-α/LPS MDSCs had reduced serum ALT and AST levels (**Figures 5C, D**).

To further investigate the therapeutic effect of the post-treatment of MDSC, TNF-α/LPS MDSCs were adoptively transferred to the mice at 2 h after APAP injection. We also compared the therapeutic effect of the antidote, N-acetylcysteine (NAC), and the adoptive transfer of TNF-α/LPS MDSCs. Although the early injection of N-acetylcysteine to APAP-treated mice remarkably reduced the hepatotoxicity of the overdose of APAP (**Supplementary Figure 4**), the post-treatment of N-acetylcysteine had no therapeutic effect. Compared to N-acetylcysteine, the post-treatment of TNF-α/LPS MDSCs had a better therapeutic effect in reducing the serum ALT levels and mortality (**Figures 4E, F**). These data indicated that TNF-α/LPS MDSCs were the most potent BM-MDSCs to protect the mice from APAP-induced liver failure.

### iNOS Is the Key Molecule for the Protection of TNF-α/LPS MDSCs Against Lethal APAP challenge

**Figure 6:**
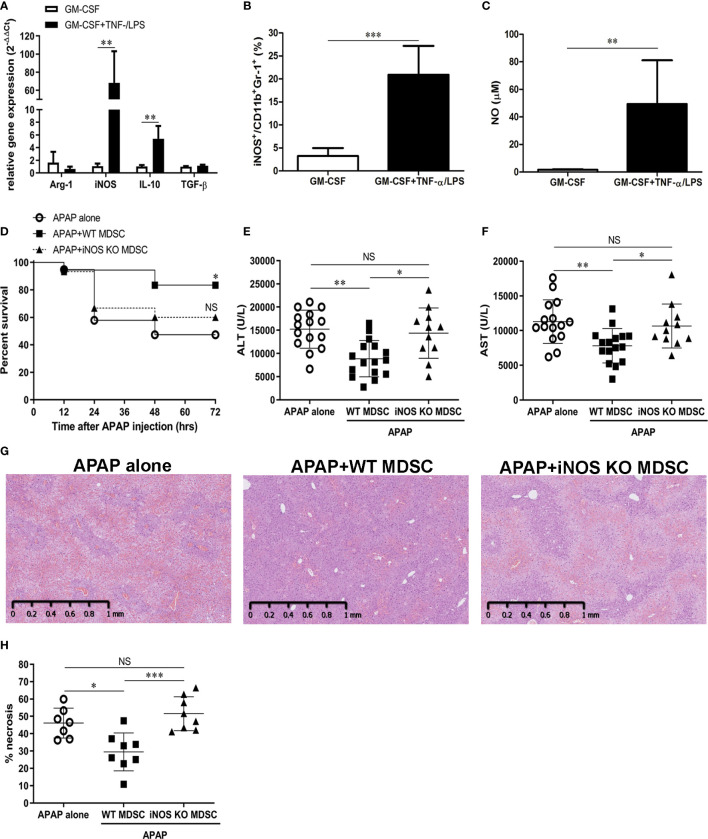
TNF-α/lipopolysaccharide (LPS) myeloid-derived suppressor cells (MDSCs) ameliorate acetaminophen (APAP)-induced liver injury through iNOS **(A)** mRNA expression of *in vitro*-generated MDSCs cultured with GM-CSF alone or combined with TNF-α/LPS. (n=6),**P < 0.01, Mann-Whitney test. **(B)** iNOS expression in MDSCs was measured by flow cytometry(n=8), ***P < 0.001, Mann-Whitney test. **(C)** NO production in the culture media of MDSCs (n=6), **P < 0.01, Mann-Whitney test. **(D)** BALB/c mice were adoptively transferred with WT or iNOS KO TNF-α/LPS MDSCs just after APAP challenge. The survival of mice was plotted (n=17-20). *P < 0.05, NS, no significance, Log-rank test. **(E)** The serum ALT at 12 h after APAP challenge (n=11-16). **(F)** The serum AST at 12 h after APAP challenge (n=11-16).*P < 0.05, **P < 0.01, NS, no significance. Kruskal-Wallis test. Data were expressed as mean ± SD. **(G)** Representitive of liver histological stain of mice with indicative treatment. **(H)** The quatification of liver necrosis (n=7-8). *P < 0.05, ***P < 0.001, NS, no significance. Kruskal-Wallis test. Data were expressed as mean ± SD.

We next analyzed the phenotypes of TNF-α/LPS MDSCs to explore the possible molecular mechanisms of this protection. The relative gene expression data indicated that both iNOS and IL-10 increased in TNF-α/LPS MDSCs with the iNOS to be the significant one (**Figure 6A**). Similary, the protein level of iNOS was significantly higher in TNF-α/LPS MDSCs than BM-MDSCs cultured with GM-CSF alone (GM-CSF MDSC) as evaluated by flow cytometry (**Figure 6B**). To measure the functional activity of iNOS in TNF-α/LPS MDSC, we examined the concentration of nitric oxide (NO), which is the end product of iNOS, in the cell culture media. The concentration of NO was significantly higher in TNF-α/LPS MDSCs than GM-CSF MDSCs (**Figure 6C**). We then adoptively transferred TNF-α/LPS MDSCs derived from either wild type (WT TNF-α/LPS MDSCs) or iNOS knockout mice (iNOS KO TNF-α/LPS MDSCs) into APAP-treated mice to investigate the role of iNOS. As expected, without the expression of iNOS, the protection ability of TNF-α/LPS MDSCs was lost (**Figures 6D–H**).

### TNF-α/LPS MDSCs Reduced Neutrophil Infiltration Into the Liver After APAP Challenge

**Figure 7:**
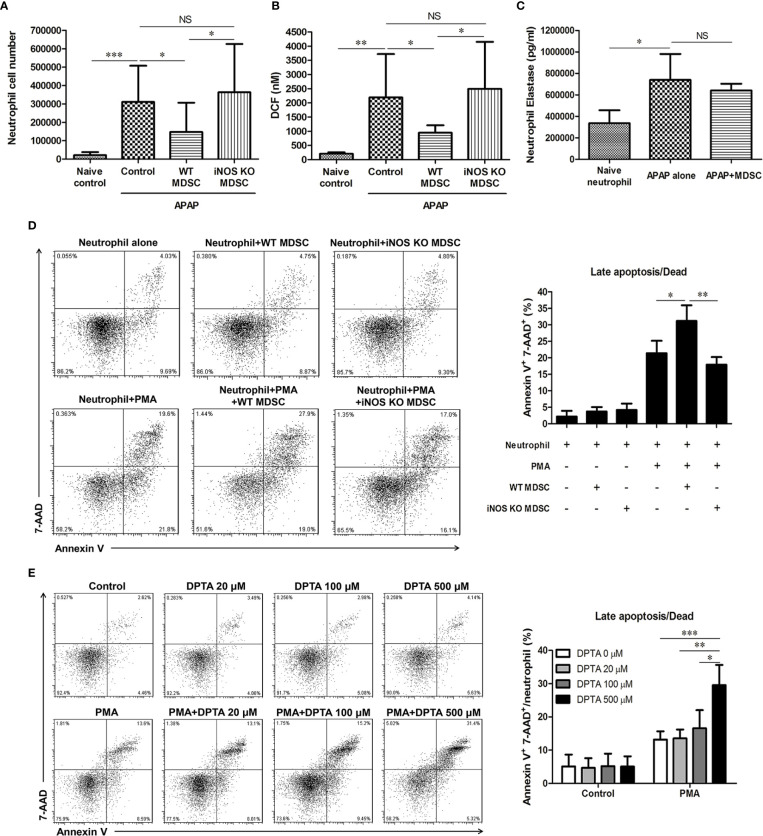
Nitric oxide promotes the apoptosis of activated neutrophil **(A)** The hepatic cell numbers of CD11b^+^Ly6G^+^ neutrophil at 6 h after acetaminophen (APAP) injection (n=6–8). *P < 0.05, ***P < 0.001, NS, no significance, Kruskal-Wallis test. **(B)** Total ROS/RNS levels in liver tissue are represented by DCF concentration. *P < 0.05, **P < 0.01, NS, no significance, Kruskal-Wallis test. **(C)** Hepatic neutrophils from the mice injected with APAP alone or combined with adoptive transfer of TNF-α/lipopolysaccharide (LPS) MDSC were isolated to measure the levels of elastase (n=6 per group). Naïve neutrophils were the blood neutrophils of healthy mice (n=3). *P < 0.05, NS, no significance, Kruskal-Wallis test. For neutrophil apoptosis assessment, 1×10^5^ neutrophils were cultured with MDSCs in equal numbers **(D)** or DPTA **(E)** for 3 h (n=9 per group). 50nM of PMA was added for neutrophil activation. The apoptosis of neutrophil was analyzed by flow cytometry. *P < 0.05, **P < 0.01, ***P < 0.001,. Kruskal-Wallis test. Data were expressed as mean ± SD.

To investigate the possible underlying mechanisms by which TNF-α/LPS MDSCs reduced AILI, we analyzed the infiltration of leukocytes in the liver 6 h after APAP challenge to clarify the immune responses at the early stage of AILI. The results showed that mice adoptively transferred with WT TNF-α/LPS MDSCs had a significant decline in neutrophil numbers, while the mice with APAP challenge alone had a large number of neutrophil infiltration in the liver. Moreover, adoptively transferring iNOS KO TNF-α/LPS MDSCs to APAP-treated mice did not decrease neutrophil infiltration (**Figure 7A**). Consistent with the reduced infiltration of neutrophil, the levels of ROS and RNS, the oxidants produced by neutrophils, were significantly decreased in the liver of TNF-α/LPS MDSC-treated mice (**Figure 7B**). We further examined the phenotypes of intrahepatic neutrophil in AILI, and it showed that the intra-hepatic neutrophil had no immunosuppressive ability (**Supplementary Figure 5**) but had increased elastase (**Figure 7C**), which has been proved to amplify liver injury in AILI (6). Moreover, the adoptive transfer of TNF-α/LPS MDSCs did not reduce neutrophil elastase (**Figure 7C**). These data suggested that the adoptive transfer of TNF-α/LPS MDSCs ameliorated AILI not by decreasing the elastase expression per neutrophil but by reducing the cell number of neutrophil and its oxidants in the liver.

### Nitric Oxide Promotes the Apoptosis of Activated Neutrophil

Since the adoptive transfer of wild type MDSC but not iNOS KO MDSC reduced the infiltration of neutrophil in the liver, it is possible that the nitric oxide (NO) released from MDSC could induce neutrophil apoptosis and reduce the liver damage. We found that wild type MDSC but not iNOS KO MDSC, could promote the apoptosis of activated neutrophil (**Figure 7D**). To further verify the effect of NO on neutrophil apoptosis, we cultured neutrophil in the presence of DPTA NONOate, the NO donor. Similar to the results of the co-culture of neutrophil and wild type MDSC, the addition of DPTA NONOate also increased the apoptosis of phorbol 12-myristate 13-acetate (PMA)-stimulated neutrophil (**Figure 7E**). These data suggested that TNF-α/LPS MDSCs could induce the apoptosis of activated neutrophil through iNOS.

### *In vitro* Generation of Human MDSCs from PBMCs

**Figure 8:**
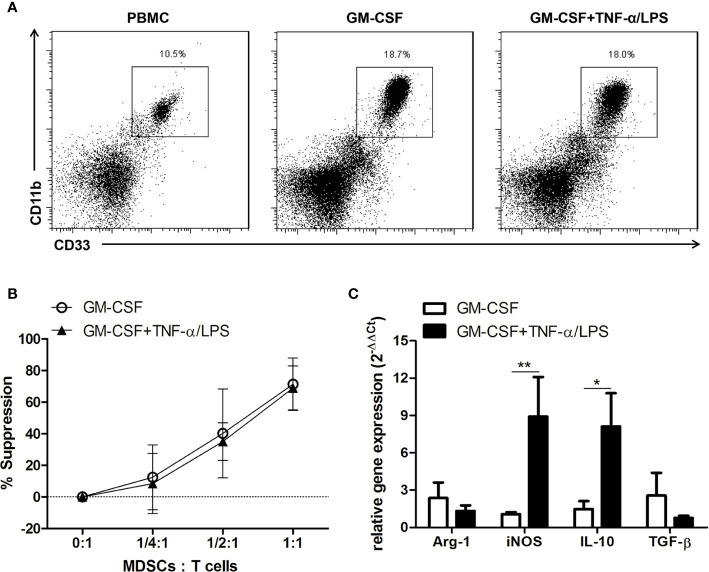
TNF-α and lipopolysaccharide (LPS) increase the mRNA expression of iNOS and IL-10 in *in vitro*-generated human myeloid-derived suppressor cells (MDSCs). **(A)** Human CD11b^+^CD33^+^ MDSCs generated from PBMCs. **(B)** The suppressive abilities of human MDSCs. **(C)** mRNA expression of human MDSCs. *P < 0.05, **P < 0.01, n=5 for each group, Mann-Whitney test. Data were expressed as mean ± SD.

The protective ability of TNF-α/LPS MDSCs as shown above highlights the possible therapeutic role of MDSCs in patients with APAP-induced acute liver failure. We therefore investigated the possibility of generation of human MDSCs for this therapeutic purpose. We cultured PBMCs isolated from healthy donors with GM-CSF alone or together with TNF-α plus LPS. After four-day culture, the percentages of MDSCs (CD11b^+^CD33^+^ myeloid cells) in the cultures were increased (**Figure 8A**). Both CD33^+^ cells cultured with GM-CSF alone or together with TNF-α plus LPS had similar potent suppressive abilities (**Figure 8B**). Moreover, similar to the mouse model, TNF-α plus LPS treatment could significantly increase the mRNA expression of iNOS and IL-10 (**Figure 8C**). Therefore, these results showed the TNF-α plus LPS together with GM-CSF could generate potent MDSCs with high iNOS expression from PBMCs and could possibly be useful for the treatment of patients with APAP-induced acute liver failure.

## Discussion

MDSCs could regulate both innate and adaptive immune responses through various suppressive mechanisms to reduce inflammation and limit tissue damage (15, 20). In our study, it is clear that intrahepatic MDSCs increased after the sublethal challenge of APAP and were responsible for the protection against subsequent lethal challenge. In addition, adoptive transfer of TNF-α/LPS MDSCs could efficiently rescue mice from mortality induced by APAP. These results are compatible with the relationship of MDSCs to the inflammations in terms of tissue protection. Moreover, we found that TNF-α/LPS MDSCs could promote the apoptosis of activated neutrophil through NO, and then decrease the intrahepatic infiltration of neutrophil resulting in ameliorating liver damage. To further investigate the possibility of *in vitro* generation of human MDSCs from PBMCs, we had shown MDSCs generated from PBMC with the help of TNF-α/LPS had similar phenotypes compatible to mouse TNF-α/LPS MDSCs. These results do not only highlight the protective role of MDSC in AILI and the importance of iNOS, but also provide a therapeutic strategy for rescuing this AILI-induced mortality by MDSC cell therapy.

The role of neutrophils in AILI is intriguing. The activation of neutrophil could propagate the sterile inflammation and aggravates liver injury (6, 11). Evidence had shown intrahepatic activated neutrophils could release elastase, MMP-9, and ROS and then exacerbate APAP hepatotoxicity (6, 13). By reducing the hepatic infiltration of neutrophil either by blocking CXCR2, TLR-9, or HMGB-1, it significantly decreased APAP hepatotoxicity in mice (6, 11, 12). However, the activation of neutrophil was also found in the resolution phase of AILI both in mouse and humans (35). In addition, a recent study indicated that these neutrophils could promote the phenotypic conversion of pro-resolving macrophages to facilitate tissue repair (36). These studies suggested that neutrophils might help to limit the further injury and facilitate the resolution in the late phase of AILI. In our study, we demonstrated that the intrahepatic CD11b^+^Ly6G^+^ neutrophils increased at the early stage of AILI expressed elastase and were not suppressors. These results were compatible the damaging role of neutrophil at the early phase of AILI. Furthermore, in our analysis, the CD11b^+^Gr-1^+^ cells were also increased in the liver in the later stage of AILI. On the other hand, it was already known a subset of pathologically activated neutrophils, named as PMN-MDSCs share morphological and phenotypic features with neutrophils but with immune-suppression abilities (37). In our further analysis, we had showed these late-increased CD11b^+^Gr-1^+^ cells were with immunosuppression ability and a portion of these cells were regarded as PMN-MDSCs, the pathologically activated neutrophils. Therefore, our observation was also compatible with previous studies that activated neutrophil also increased in the resolution phase of AILI. Taken together, our studies suggested that in the early phase the activation of neutrophil could aggravates tissue injury, but in the late phase, the pathologically activated neutrophils, PMN-MDSCs, could on the contrary limit the inflammation and facilitate the tissue repair. Till now, the only antidote for AILI is N-acetylcysteine but with moderate efficacy. It is becasue the therapeutic effect of N-acetylcysteine is tranisent and only in the very early phase of APAP due to their moderate effect on the injury phase of AILI. Based on our results, we then proposed another treatment strategy by transferring the MDSCs to inhibit the subsequent immune mediated tissue injury and to facilitate recovery in AILI.

iNOS is one of the suppressive molecules of MDSCs which can inhibit T cell proliferation and activation (17, 38). In our study, we extended the suppression mechanisms of iNOS and clearly demonstrated that iNOS was the key molecule in this MDSC-mediated protection by promoting the apoptosis of activated neutrophil through NO production. Supporting to our results, it had already been shown that incubation with a higher concentration of NO could increase neutrophil apoptosis (39, 40). Therefore, by the help of NO, MDSCs could reduce excess inflammation and ameliorate APAP hepatotoxicity by reducing the infiltration of activated neutrophil through promoting apoptosis of these neutrophils. Moreover, because MDSC did not promote the apoptosis of non-activated neutrophils, the abilities of neutrophil in the resolution of inflammation and tissue repair might be preserved.

Both Con A and LPS/D-GalN could induce acute liver injury in mice through the activation of T cell and macrophage respectively without directly inducing hepatotoxicity (32, 33). Taking the advange of the pure immune-mediated liver damage by Con A and LPS/D-GalN, we then clarified the immune suppression role of MDSC induced by the sublethal challenge of APAP by a subsequent lethal challenge of Con A or LPS/D-GalN. Our results clearly demonstrated the protective role of MDSC by their immune-suppression effects.

In mice, CD11b^+^Gr-1^+^ cells are defined as total MDSCs, including PMN-MDSCs (CD11b^+^Gr-1^hi^Ly6C^low/int^Ly6G^+^) and M-MDSCs (CD11b^+^Gr-1^int^Ly6C^hi^Ly6G^–^) (41). The two subsets of MDSCs could not be selectively depleted by monoclonal antibodies. However, the anti-Gr1 antibodies could only deplete all the MDSCs *in vivo* (**Supplementary Figure 2**). Furthermore, both CD11b^+^Gr-1^hi^ and CD11b^+^Gr-1^int^ cells were increased after APAP challenge (**Supplementary Figure 6**). The *in vitro*-generated BM-MDSCs also consist of both CD11b^+^Gr-1^hi^ and CD11b^+^Gr-1^int^ cells and the iNOS expression similarly increased in both subsets of BM-MDSCs (**Supplementary Figure 7**). Therefore, we did not separate further into PMN-MDSC and M-MDSC subgroups, but instead used the MDSCs as the target of this study.

Taking advantage of the potent immune regulatory abilities, MDSCs have been proposed as cell therapies to treat immune-mediated diseases. Using different cytokines, such as IL-6, IL-13, or LPS, it could generate BM-MDSCs *in vitro* and successfully treated diseases *in vivo*, like graft-versus-host disease, skin transplantation and pancreatic islet transplantation (22, 23, 42, 43). Based on these successful studies of MDSC-based cell therapies, we then investigated the therapeutic role of MDSCs in AILI. As shown in our studies, the adoptively transferred TNF-α/LPS MDSCs did really reduce liver injury and rescue mice from the mortality. Moreover, we also successfully generated MDSCs from human PBMCs with similar phenotype to murine TNF-α/LPS MDSCs. Therefore, this MDSC-based cell therapy could be possibly a promising treatment strategy for patients with APAP-induced liver failure.

## Data Availability Statement

The original contributions presented in the study are included in the article/**Supplementary Material**. Further inquiries can be directed to the corresponding author.

## Ethics Statement

The studies involving human participants were reviewed and approved by Chang Gung Medical Foundation Institutional Review Board. The patients/participants provided their written informed consent to participate in this study. The animal study was reviewed and approved by the institutional animal ethics committee of the laboratory animal center of Chang Gung Medical Foundation.

## Author Contributions

C-YH: acquisition of data, analysis and interpretation of data, drafting of the manuscript, statistical analysis. Y-CL: study concept and design, critical revision of the manuscript for important intellectual content, obtained funding, administrative support. L-YC: study concept and design, critical revision of the manuscript for important intellectual content. S-KH: acquisition of data, administrative and material support. C-HH: critical revision of the manuscript for important intellectual content, obtained funding. C-KY: critical revision of the manuscript for important intellectual content, obtained funding. C-TH: critical revision of the manuscript for important intellectual content, obtained funding, administrative and material support. C-YL: study concept and design, critical revision of the manuscript for important intellectual content, drafting of the manuscript, obtained funding, administrative support, study supervision. All authors contributed to the article and approved the submitted version.

## Funding

This work was supported by CMRPG3G0531, CMRPG3G0532, CMRPG3G0533; CMRPG3F0592, CMRPG3F0593; CMRPG392133; CORPG3I0071; CMRPG3J0791 from Medical Research Project Fund, Chang Gung Memorial Hospital, and MOST 104-2314-B-182A-016- from Ministry of Science and Technology, Taiwan.

## Conflict of Interest

The authors declare that the research was conducted in the absence of any commercial or financial relationships that could be construed as a potential conflict of interest.

## References

[1] Jaeschke H Acetaminophen: Dose-Dependent Drug Hepatotoxicity and Acute Liver Failure in Patients. Dig Dis (2015) 33(4):464–71. 10.1159/000374090 PMC4520394 26159260

[2] Lee WM Acetaminophen (APAP) hepatotoxicity-Isn’t it time for APAP to go away? J Hepatol (2017) 67(6):1324–31. 10.1016/j.jhep.2017.07.005 PMC5696016 28734939

[3] Saito C Zwingmann C Jaeschke H Novel mechanisms of protection against acetaminophen hepatotoxicity in mice by glutathione and N-acetylcysteine. Hepatology (2010) 51(1):246–54. 10.1002/hep.23267 PMC2977522 19821517

[4] McGill MR Sharpe MR Williams CD Taha M Curry SC Jaeschke H The mechanism underlying acetaminophen-induced hepatotoxicity in humans and mice involves mitochondrial damage and nuclear DNA fragmentation. J Clin Invest (2012) 122(4):1574–83. 10.1172/JCI59755 PMC3314460 22378043

[5] Krenkel O Mossanen JC Tacke F Immune mechanisms in acetaminophen-induced acute liver failure. Hepatobiliary Surg Nutr (2014) 3(6):331–43. 10.3978/j.issn.2304-3881.2014.11.01 PMC4273118 25568858

[6] Huebener P Pradere JP Hernandez C Gwak GY Caviglia JM Mu X The HMGB1/RAGE axis triggers neutrophil-mediated injury amplification following necrosis. J Clin Invest (2015) 125(2):539–50. 10.1172/JCI76887 PMC4319429 25562324

[7] Mossanen JC Krenkel O Ergen C Govaere O Liepelt A Puengel T Chemokine (C-C motif) receptor 2-positive monocytes aggravate the early phase of acetaminophen-induced acute liver injury. Hepatology (2016) 64(5):1667–82. 10.1002/hep.28682 27302828

[8] Maher JJ DAMPs ramp up drug toxicity. J Clin Invest (2009) 119(2):246–9. 10.1172/JCI38178 PMC2631305 19244605

[9] Zhang C Feng J Du J Zhuo Z Yang S Zhang W Macrophage-derived IL-1alpha promotes sterile inflammation in a mouse model of acetaminophen hepatotoxicity. Cell Mol Immunol (2018) 15(11):973–82. 10.1038/cmi.2017.22 PMC6207754 28504245

[10] Graubardt N Vugman M Mouhadeb O Caliari G Pasmanik-Chor M Reuveni D Ly6C(hi) Monocytes and Their Macrophage Descendants Regulate Neutrophil Function and Clearance in Acetaminophen-Induced Liver Injury. Front Immunol (2017) 8:626. 10.3389/fimmu.2017.00626 28620383 PMC5451509

[11] Marques PE Amaral SS Pires DA Nogueira LL Soriani FM Lima BH Chemokines and mitochondrial products activate neutrophils to amplify organ injury during mouse acute liver failure. Hepatology (2012) 56(5):1971–82. 10.1002/hep.25801 22532075

[12] Marques PE Oliveira AG Pereira RV David BA Gomides LF Saraiva AM Hepatic DNA deposition drives drug-induced liver injury and inflammation in mice. Hepatology (2015) 61(1):348–60. 10.1002/hep.27216 24824608

[13] Kojo K Ito Y Eshima K Nishizawa N Ohkubo H Yokomizo T BLT1 signalling protects the liver against acetaminophen hepatotoxicity by preventing excessive accumulation of hepatic neutrophils. Sci Rep (2016) 6:29650. 10.1038/srep29650 27404729 PMC4939602

[14] Raevens S Van Campenhout S Debacker PJ Lefere S Verhelst X Geerts A Combination of sivelestat and N-acetylcysteine alleviates the inflammatory response and exceeds standard treatment for acetaminophen-induced liver injury. J Leukoc Biol (2020) 107(2):341–55. 10.1002/JLB.5A1119-279R 31841237

[15] Ostrand-Rosenberg S Sinha P Myeloid-derived suppressor cells: linking inflammation and cancer. J Immunol (2009) 182(8):4499–506. 10.4049/jimmunol.0802740 PMC2810498 19342621

[16] Ioannou M Alissafi T Lazaridis I Deraos G Matsoukas J Gravanis A Crucial role of granulocytic myeloid-derived suppressor cells in the regulation of central nervous system autoimmune disease. J Immunol (2012) 188(3):1136–46. 10.4049/jimmunol.1101816 22210912

[17] Kumar V Patel S Tcyganov E Gabrilovich DI The Nature of Myeloid-Derived Suppressor Cells in the Tumor Microenvironment. Trends Immunol (2016) 37(3):208–20. 10.1016/j.it.2016.01.004 PMC4775398 26858199

[18] Gabrilovich DI Nagaraj S Myeloid-derived suppressor cells as regulators of the immune system. Nat Rev Immunol (2009) 9(3):162–74. 10.1038/nri2506 PMC2828349 19197294

[19] Bronte V Brandau S Chen SH Colombo MP Frey AB Greten TF Recommendations for myeloid-derived suppressor cell nomenclature and characterization standards. Nat Commun (2016) 7:12150. 10.1038/ncomms12150 27381735 PMC4935811

[20] Nagaraj S Youn JI Gabrilovich DI Reciprocal relationship between myeloid-derived suppressor cells and T cells. J Immunol (2013) 191(1):17–23. 10.4049/jimmunol.1300654 23794702 PMC3694485

[21] Hoechst B Voigtlaender T Ormandy L Gamrekelashvili J Zhao F Wedemeyer H Myeloid derived suppressor cells inhibit natural killer cells in patients with hepatocellular carcinoma *via* *the* NKp30 receptor. Hepatology (2009) 50(3):799–807. 10.1002/hep.23054 19551844 PMC6357774

[22] Highfill SL Rodriguez PC Zhou Q Goetz CA Koehn BH Veenstra R Bone marrow myeloid-derived suppressor cells (MDSCs) inhibit graft-versus-host disease (GVHD) *via* *an* arginase-1-dependent mechanism that is up-regulated by interleukin-13. Blood (2010) 116(25):5738–47. 10.1182/blood-2010-06-287839 PMC3031417 20807889

[23] Marigo I Bosio E Solito S Mesa C Fernandez A Dolcetti L Tumor-induced tolerance and immune suppression depend on the C/EBPbeta transcription factor. Immunity (2010) 32(6):790–802. 10.1016/j.immuni.2010.05.010 20605485

[24] Messmann JJ Reisser T Leithauser F Lutz MB Debatin KM Strauss G In vitro-generated MDSCs prevent murine GVHD by inducing type 2 T cells without disabling antitumor cytotoxicity. Blood (2015) 126(9):1138–48. 10.1182/blood-2015-01-624163 26185131

[25] Zahid MF Lazarus HM Ringden O Barrett JA Gale RP Hashmi SK Can we prevent or treat graft-versus-host disease with cellular-therapy? Blood Rev (2020) 43:100669. 10.1016/j.blre.2020.100669 32089398

[26] Du K Williams CD McGill MR Jaeschke H Lower susceptibility of female mice to acetaminophen hepatotoxicity: Role of mitochondrial glutathione, oxidant stress and c-jun N-terminal kinase. Toxicol Appl Pharmacol (2014) 281(1):58–66. 10.1016/j.taap.2014.09.002 25218290 PMC4362889

[27] Watanabe H Ohtsuka K Kimura M Ikarashi Y Ohmori K Kusumi A Details of an isolation method for hepatic lymphocytes in mice. J Immunol Methods (1992) 146(2):145–54. 10.1016/0022-1759(92)90223-G 1531671

[28] Livak KJ Schmittgen TD Analysis of relative gene expression data using real-time quantitative PCR and the 2(-Delta Delta C(T)) Method. Methods (2001) 25(4):402–8. 10.1006/meth.2001.1262 11846609

[29] Lechner MG Liebertz DJ Epstein AL Characterization of cytokine-induced myeloid-derived suppressor cells from normal human peripheral blood mononuclear cells. J Immunol (2010) 185(4):2273–84. 10.4049/jimmunol.1000901 PMC2923483 20644162

[30] Ben-Shachar R Chen Y Luo S Hartman C Reed M Nijhout HF The biochemistry of acetaminophen hepatotoxicity and rescue: a mathematical model. Theor Biol Med Model (2012) 9:55. 10.1186/1742-4682-9-55 23249634 PMC3576299

[31] Shayiq RM Roberts DW Rothstein K Snawder JE Benson W Ma X Repeat exposure to incremental doses of acetaminophen provides protection against acetaminophen-induced lethality in mice: an explanation for high acetaminophen dosage in humans without hepatic injury. Hepatology (1999) 29(2):451–63. 10.1002/hep.510290241 9918922

[32] Tiegs G Hentschel J Wendel A A T cell-dependent experimental liver injury in mice inducible by concanavalin A. J Clin Invest (1992) 90(1):196–203. 10.1172/JCI115836 1634608 PMC443081

[33] Su GL Lipopolysaccharides in liver injury: molecular mechanisms of Kupffer cell activation. Am J Physiol Gastrointest Liver Physiol (2002) 283(2):G256–65. 10.1152/ajpgi.00550.2001 12121871

[34] Condamine T Gabrilovich DI Molecular mechanisms regulating myeloid-derived suppressor cell differentiation and function. Trends Immunol (2011) 32(1):19–25. 10.1016/j.it.2010.10.002 21067974 PMC3053028

[35] Williams CD Bajt ML Sharpe MR McGill MR Farhood A Jaeschke H Neutrophil activation during acetaminophen hepatotoxicity and repair in mice and humans. Toxicol Appl Pharmacol (2014) 275(2):122–33. 10.1016/j.taap.2014.01.004 PMC3941640 24440789

[36] Yang W Tao Y Wu Y Zhao X Ye W Zhao D Neutrophils promote the development of reparative macrophages mediated by ROS to orchestrate liver repair. Nat Commun (2019) 10(1):1076. 10.1038/s41467-019-09046-8 30842418 PMC6403250

[37] Zhou J Nefedova Y Lei A Gabrilovich D Neutrophils and PMN-MDSC: Their biological role and interaction with stromal cells. Semin Immunol (2018) 35:19–28. 10.1016/j.smim.2017.12.004 29254756 PMC5866202

[38] Gabrilovich DI Velders MP Sotomayor EM Kast WM Mechanism of immune dysfunction in cancer mediated by immature Gr-1+ myeloid cells. J Immunol (2001) 166(9):5398–406. 10.4049/jimmunol.166.9.5398 11313376

[39] Dubey M Nagarkoti S Awasthi D Singh AK Chandra T Kumaravelu J Nitric oxide-mediated apoptosis of neutrophils through caspase-8 and caspase-3-dependent mechanism. Cell Death Dis (2016) 7(9):e2348. 10.1038/cddis.2016.248 27584786 PMC5059853

[40] Fortenberry JD Owens ML Brown MR Atkinson D Brown LA Exogenous nitric oxide enhances neutrophil cell death and DNA fragmentation. Am J Respir Cell Mol Biol (1998) 18(3):421–8. 10.1165/ajrcmb.18.3.2875 9490660

[41] Gabrilovich DI Ostrand-Rosenberg S Bronte V Coordinated regulation of myeloid cells by tumours. Nat Rev Immunol (2012) 12(4):253–68. 10.1038/nri3175 PMC3587148 22437938

[42] Greifenberg V Ribechini E Rossner S Lutz MB Myeloid-derived suppressor cell activation by combined LPS and IFN-gamma treatment impairs DC development. Eur J Immunol (2009) 39(10):2865–76. 10.1002/eji.200939486 19637228

[43] Drujont L Carretero-Iglesia L Bouchet-Delbos L Beriou G Merieau E Hill M Evaluation of the therapeutic potential of bone marrow-derived myeloid suppressor cell (MDSC) adoptive transfer in mouse models of autoimmunity and allograft rejection. PLoS One (2014) 9(6):e100013. 10.1371/journal.pone.0100013 24927018 PMC4057339

