# Supplementary material for: Therapeutic Role of Inducible Nitric Oxide Synthase Expressing Myeloid-Derived Suppressor Cells in Acetaminophen-Induced Murine Liver Failure
Source: Front Immunol. 2020 Oct 29;11:574839. doi: 10.3389/fimmu.2020.574839 (PMC7673381; doi:10.3389/fimmu.2020.574839)
Supplement: Supplementary file 1 [file Table_1.docx]

Supplementary Material

# Supplementary methods

**Hepatic reduced GSH detection**

The supernatant of homogenized liver tissue was collected to measure the glutathione level by total glutathione detection kit (Enzo Life Sciences Cat# ADI-900-160). The concentration of reduced GSH was obtained by subtracting the concentration of oxidized GSSG from total GSH.

***in vitro* activity assay of arginase**

Homogenize mouse liver tissue was used to measure hepatic arginase activity by Arginase Activity Colorimetric Assay Kit (BioVision Cat# K755).

# Supplementary figures and tables

**Supplementary table. Primers for real-time PCR**

| GENE | Forward primers (5’ to 3’) | Reverse primers (5’ to 3’) |
| --- | --- | --- |
| β-actin | GCCCAGAGCAAGAGAGGTAT | CACACGCAGCTCATTGTAGA |
| ARG-1 | CAGAAGAATGGAAGAGTCAG | CAGATATGCAGGGAGTCACC |
| iNOS | ACCATGGAGCATCCCAAGTA | CCATGTACCAACCATTGAAGG |
| IL-10 | AACTGCACCCACTTCCCAGTC | CATTAAGGAGTCGGTTAGCAG |
| TGF-β | GCAACATGTGGAACTCTACCAGAA | GACGTCAAAAGACAGCCACTCA |

**Supplementary figures**

**
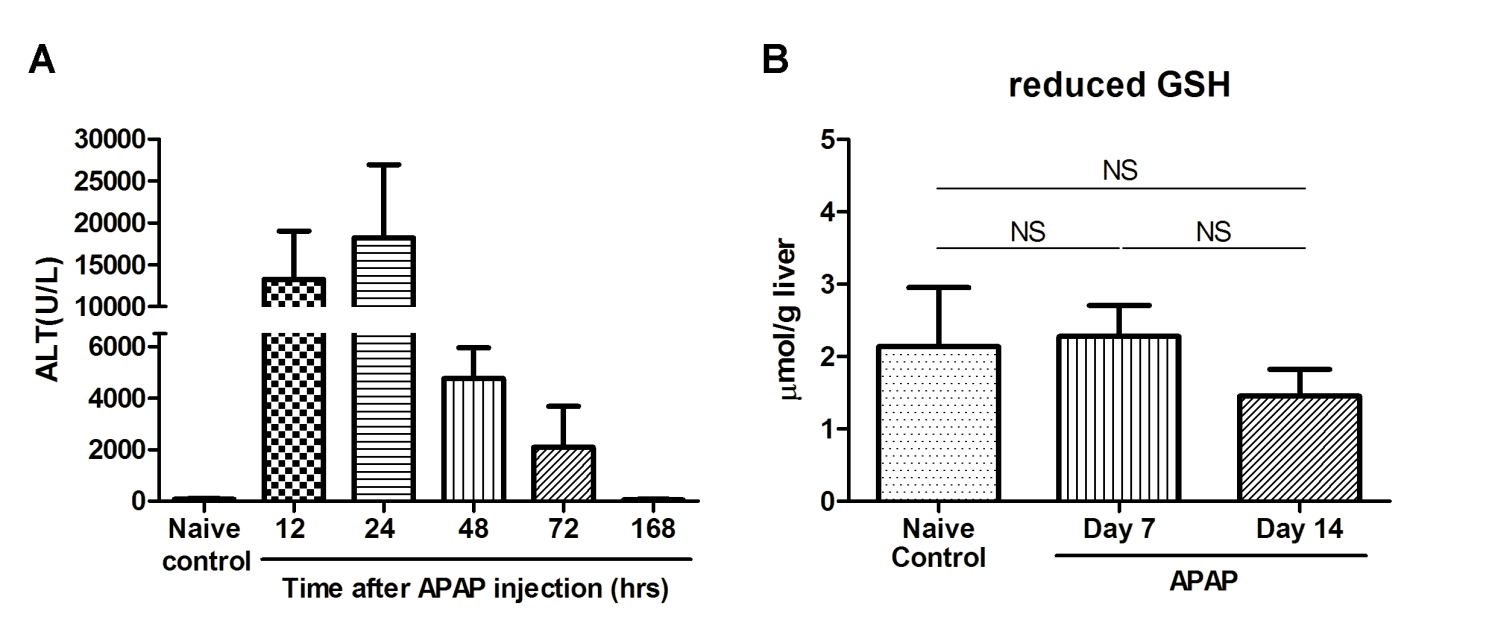
Supplementary Figure 1. Serum ALT and hepatic glutathione of sublethal APAP-treated mice. (A)** Time course of serum ALT levels of sublethal APAP-treated mice after APAP pretreatment. The serum ALT level of healthy mice is the naïve control group. **(B)** Hepatic reduced forms of glutathione (GSH) was measured on day 7 or day 14 after APAP pretreatment (n=6 per group). The naïve control group is healthy control mice. NS, no significance, Kruskal-Wallis test.

**A**

**
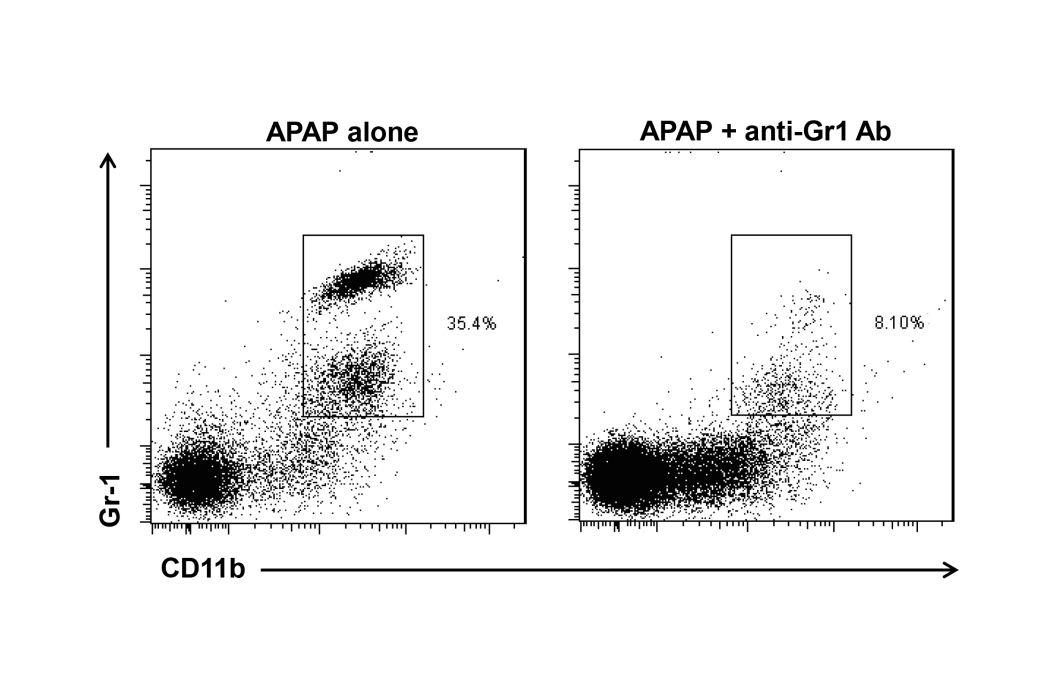
**

**B**


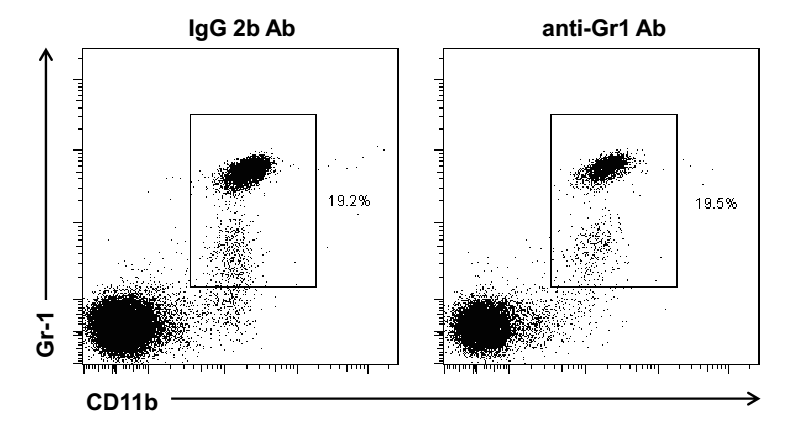


**C**


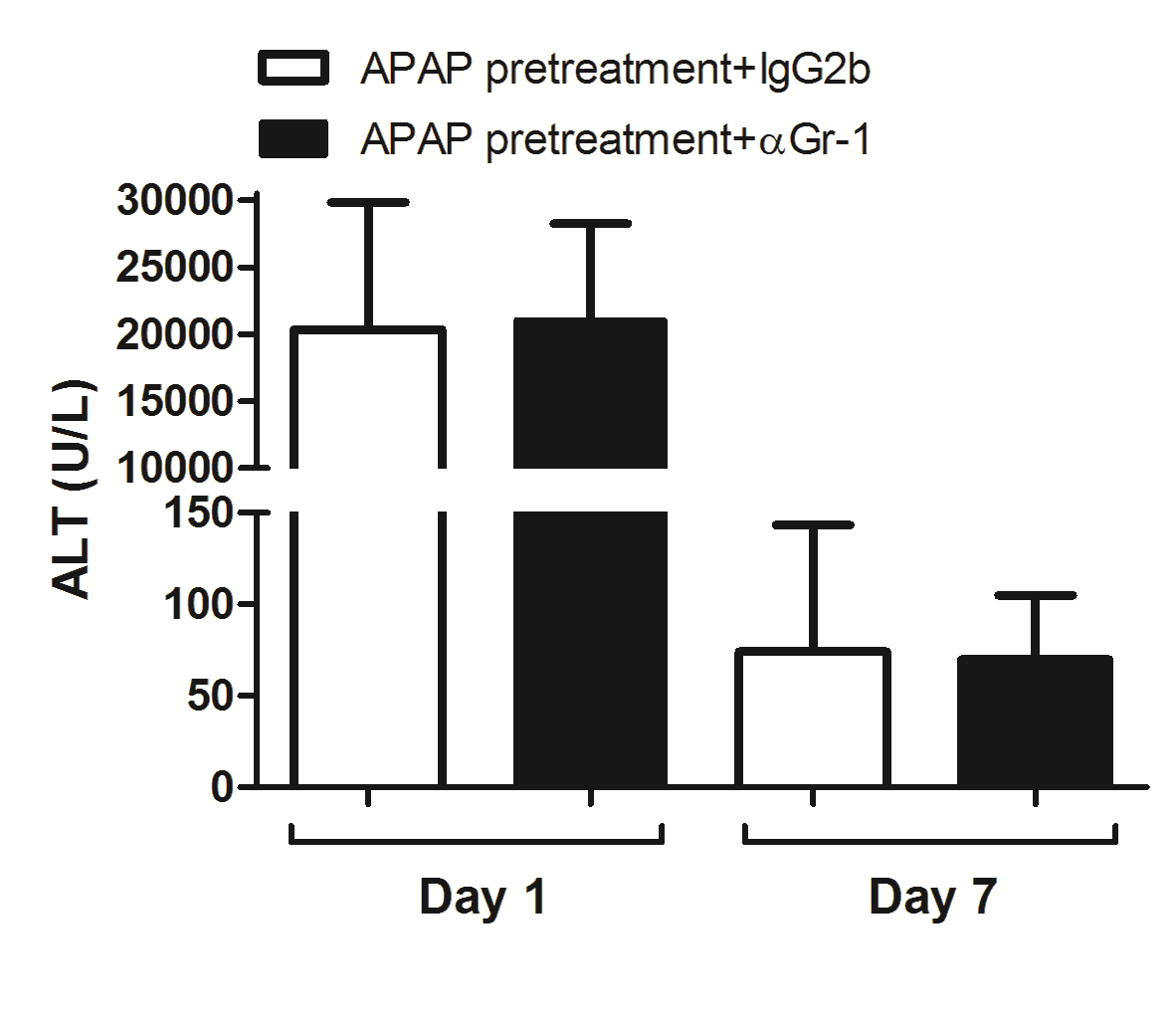


**Supplementary Figure 2. The injection of anti-Gr1 antibody on day 1 after APAP pretreatemnt decreases the percentages of CD11b^+^Gr-1^+^ cells in the liver, but does not affect the serum ALT level after APAP pretreatment. (A)** Mice were injected with anti-Gr1 antibody on day 1 after APAP pretreatment. Twenty-four hours after antibody injection, mice were sacrificed to analyze the percentages of CD11b^+^Gr-1^+^ cells in the liver by flow cytometry. **(B)** The percentages of CD11b^+^Gr-1^+^ cells in the blood on day 6 after the injection of anti-Gr1 or isotype IgG2b antibody. **(C)** The serum ALT level of mice on day 1 and day 7 after APAP pretreatment (n=8 per group). Anti-Gr1 antibody or Rat IgG2b isotype antibody was injected to the mice on day 1after APAP pretreatment.


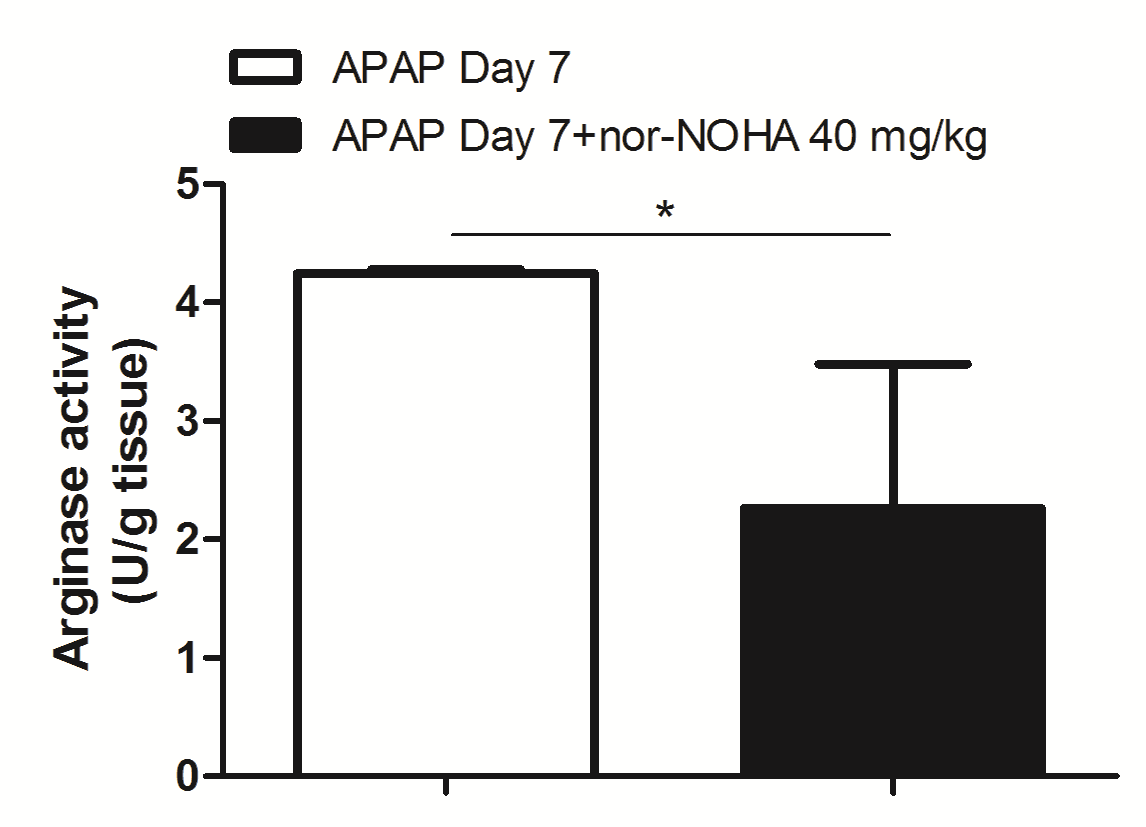


**Supplementary Figure 3. nor-NOHA reduces arginase activity in the liver.** 7 days after APAP pretreatment, mice were separated into two groups, one is the control group (APAP day 7, n=4) and the other one is the group of mice treated with nor-NOHA, an arginase inhibitor (APAP day 7+ nor-NOHA 40 mg/kg, n=4). Arginase activities of the livers were measured. **P* < 0.05, Mann-Whitney test.


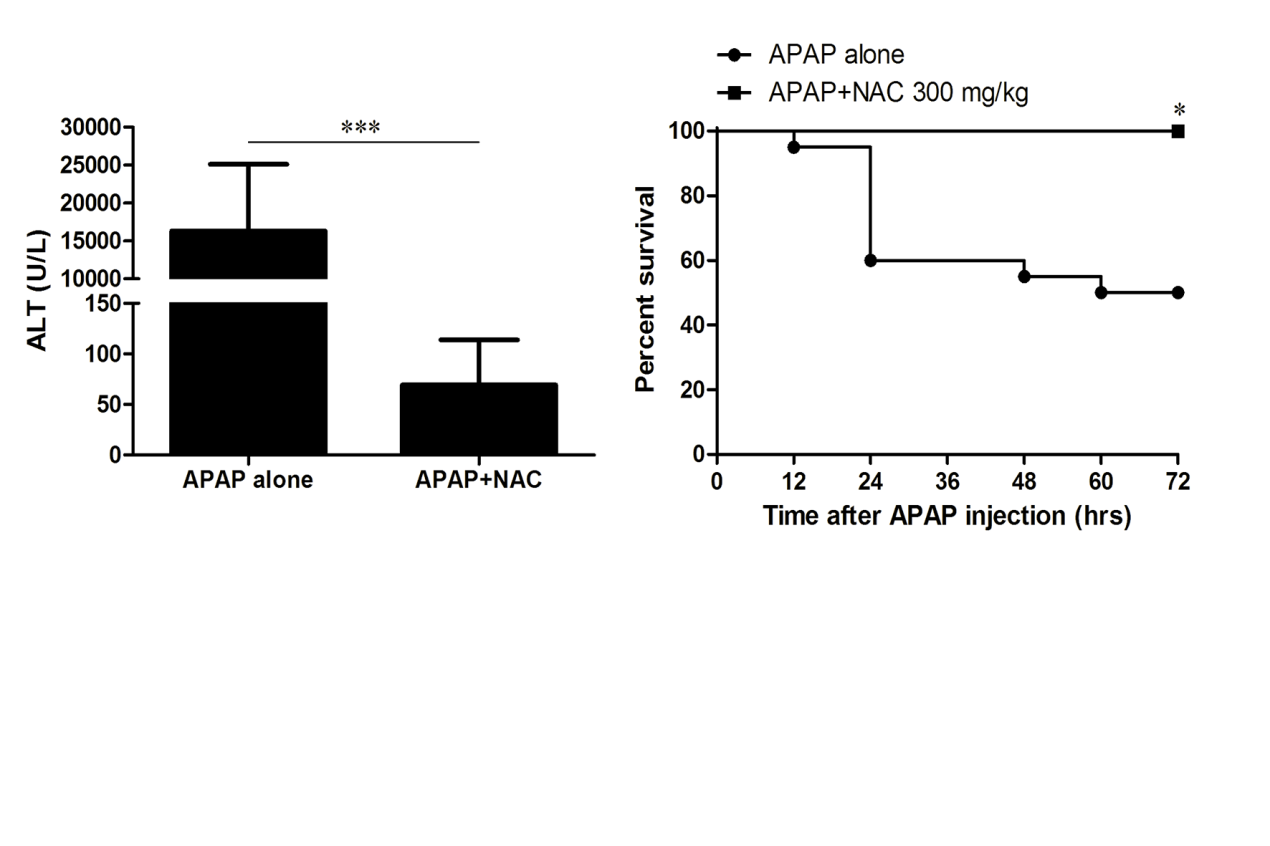


**Supplementary Figure 4. The early administration of N-acetylcysteine has the best efficacy to reduce liver injury and protect mice from death.** 300 mg/kg of N-acetylcysteine (NAC) was intraperitoneally injected into the mice 30 minutes after APAP challenge. For serum ALT measurement, the blood was collected at 12 hours after APAP challenge, ****P* < 0.001, Mann-Whitney test. The survival curves of APAP-treated mice was plotted (n=8 per group). **P* < 0.05, Log-rank Test.


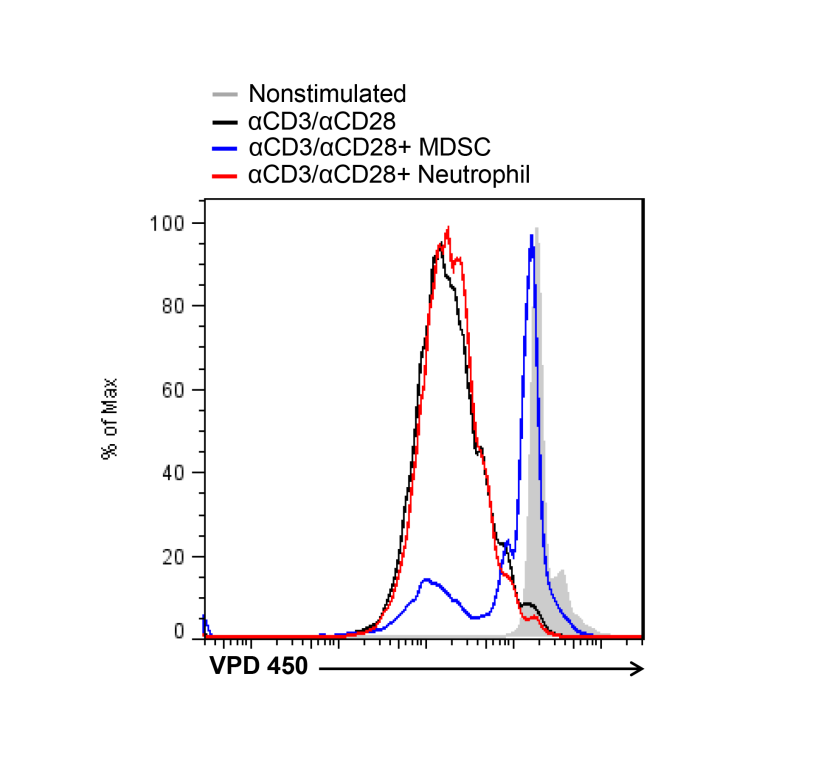


**Supplementary Figure 5. Neutrophils isolated from the liver at 6 hours after APAP treatment have no suppressive ability.** Ly6G^+^ neutrophils and CD11b^+^Gr-1^+^ MDSCs were isolated from the livers at 6 hours or 7 days after APAP treatment respectively. Neutrophils and MDSCs were cultured with CD8^+^ T cells at a ratio of 2:1 (Neutrophils or MDSCs : CD8 T cells). CD8^+^ T cells were labeled with Violet Proliferation Dye 450 (VPD450) and stimulated with anti-CD3/CD28 antibodies. The proliferation of CD8^+^ T cell was analyzed by flow cytometry.


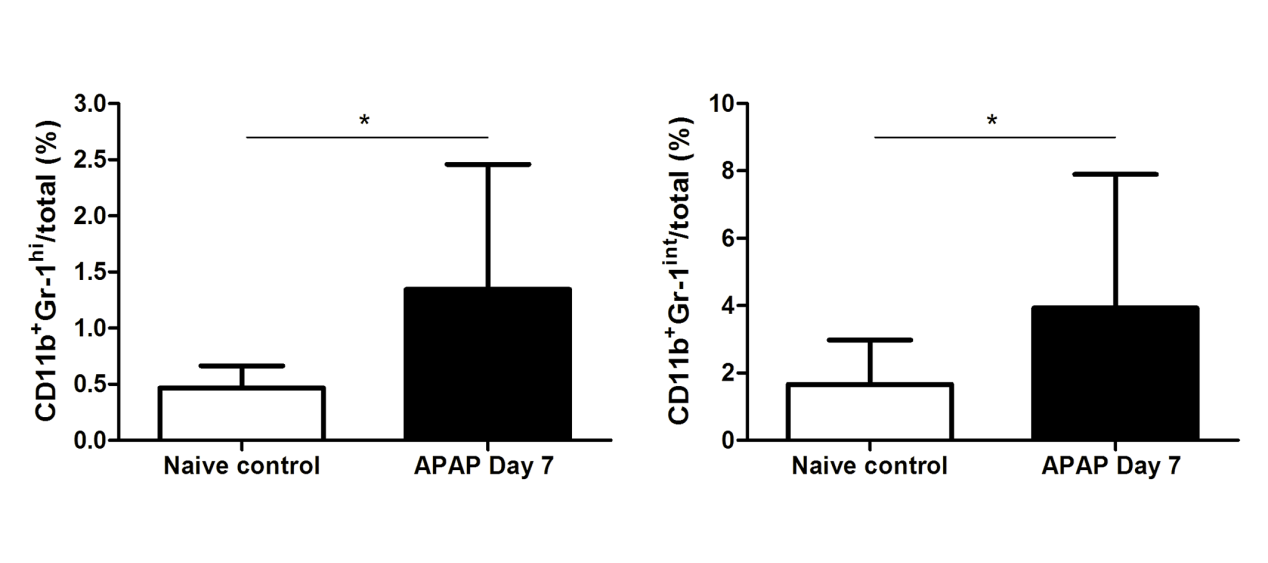


**Supplementary Figure 6. The percentages of CD11b^+^Gr-1^hi^ cells and CD11b^+^Gr-1^int^ cells in the liver of APAP-treated mice.** The percentages of CD11b^+^Gr-1^hi^ and CD11b^+^Gr-1^int^ cells in the liver at 7 days after APAP treatment. The Naive control group was healthy mice without any treatment (n=12 per group). **P* < 0.05, Mann-Whitney test.


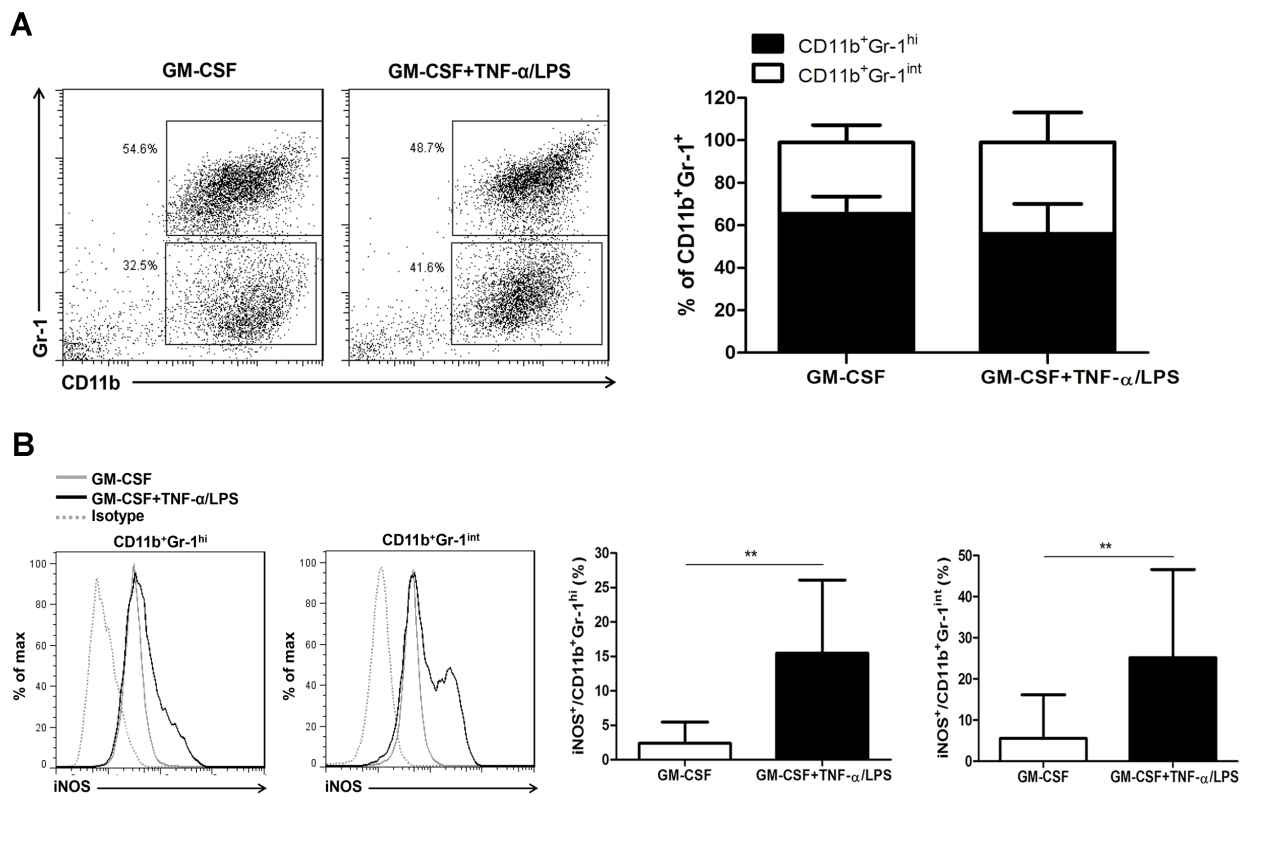


**Supplementary Figure 7. The expression of iNOS increases in both CD11b^+^Gr-1^hi^ cells and CD11b^+^Gr-1^int^ cells of TNF-α/LPS MDSCs. (A)** The percentages of CD11b^+^Gr-1^hi^ and CD11b^+^Gr-1^int^ cells in the CD11b^+^Gr-1^+^ BM-MDSCs. **(B)** The expression of iNOS in the two subsets of the CD11b^+^Gr-1^+^ BM-MDSCs, (n=8 per group). ***P* < 0.01, Mann-Whitney test.
